# Exogenous Gibberellic Acid or Dilute Bee Honey Boosts Drought Stress Tolerance in *Vicia faba* by Rebalancing Osmoprotectants, Antioxidants, Nutrients, and Phytohormones

**DOI:** 10.3390/plants10040748

**Published:** 2021-04-11

**Authors:** Mostafa M. Rady, Sara H. K. Boriek, Taia A. Abd El-Mageed, Mohamed A. Seif El-Yazal, Esmat F. Ali, Fahmy A. S. Hassan, Abdelsattar Abdelkhalik

**Affiliations:** 1 Botany Department, Faculty of Agriculture, Fayoum University, Fayoum 63514, Egypt; sarahamdy396@gmail.com (S.H.K.B.); maa04@fayoum.edu.eg (M.A.S.E.-Y.); 2 Soil and Water Science Department, Faculty of Agriculture, Fayoum University, Fayoum 63514, Egypt; taa00@fayoum.edu.eg; 3 Department of Biology, College of Science, Taif University, P.O. Box 11099, Taif 21944, Saudi Arabia; a.esmat@tu.edu.sa (E.F.A.); d.fahmy@tu.edu.sa (F.A.S.H.); 4 Horticulture Department, Faculty of Agriculture, Fayoum University, Fayoum 63514, Egypt

**Keywords:** faba bean, drought, growth and productivity, antioxidants defense system, biostimulants

## Abstract

The use of growth regulators such as gibberellic acid (GA_3_) and biostimulants, including diluted bee honey (Db-H) can improve drought tolerance in many crops, including the faba bean (*Vicia faba* L.). Db-H contains high values of osmoprotectants, mineral nutrients, vitamins, and many antioxidants making it an effective growth regulator against environmental stress effects. Therefore, the present study was planned to investigate the potential improvement in the faba bean plant performance (growth and productivity) under full watering (100% of crop evapotranspiration (ETc)) and drought stress (60% of ETc) by foliar application of GA_3_ (20 mg L^−1^) or Db-H (20 g L^−1^). The ameliorative impacts of these growth regulators on growth, productivity, physio-biochemical attributes, nutrient status, antioxidant defense system, and phytohormones were evaluated. GA_3_ or Db-H attenuated the negative influences of drought stress on cell membrane stability, ion leakage, relative water content, nutrient status, leaf pigments related to photosynthesis (chlorophylls and carotenoids), and efficiency of the photosystem II (PSII in terms of F_v_/F_m_ and performance index), thus improving faba bean growth, green pod yield, and water use efficiency. Drought stress caused an abnormal state of nutrients and photosynthetic machinery due to increased indicators of oxidative stress (malondialdehyde (MDA), hydrogen peroxide (H_2_O_2_) and superoxide (O_2_^•−^)), associated with increased osmoprotectants (proline, glycine betaine, soluble sugars, and soluble protein), non-enzymatic antioxidants (ascorbic acid, glutathione, and α-tocopherol), and enzymatic antioxidant activities (superoxide dismutase, catalase, glutathione reductase, and ascorbate peroxidase). However, foliar-applied GA_3_ or Db-H mediated further increases in osmoprotectants, antioxidant capacity, GA_3_, indole-3-acetic acid, and cytokinins, along with decreased levels of MDA and abscisic acid. These results suggest the use of GA_3_ or Db-H at the tested concentrations to mitigate drought-induced damage in bean plants to obtain satisfactory growth and productivity under a water deficit of up to 40%.

## 1. Introduction

Among the most important legume crops, the faba bean (*Vicia faba* L.) is widely cultivated around the world. Fresh pods and dry seeds are consumed worldwide for humans due to their nutritional value, which is considered among vegetables [1]. Faba bean is rich in protein (up to 35% of dry matter) [2], carbohydrates (51–68% of dry matter) [1], and mineral nutrients such as potassium (K), iron (Fe), calcium (Ca), magnesium (Mg), and zinc (Zn) [2,3].

Limited irrigation water is one of the biggest limiting factors for crop production [4,5], given that irrigated agriculture is the largest user of freshwater, with approximately 79% in Egypt and 69% worldwide of total water withdrawals [6]. Dwindling freshwater resources along with meeting the demand for food production requires increased water use efficiency (WUE) in both irrigated and rainfed agriculture [7,8]. 

Drought or water deficit directly impedes plant growth and productivity by causing loss of cell turgor and impairing mitosis that hinders cell elongation and division [9,10]. Osmotic stress is the primary signal in response to drought stress that induces abscisic acid (ABA) accumulation, which in turn, elicits several responses in plant cells [11,12]. As a secondary response, excessive formation of reactive oxygen species (ROS) such as hydroxyl radicals (OH^−^), hydrogen peroxide (H_2_O_2_), and superoxide radicals (O_2_^•−^) occurs due to drought in plant organelles like chloroplasts, mitochondria, and peroxisome [13,14]. These ROS disrupt the normal balance that exists between ROS production and scavenging [15]. This off-balance (due to excessive formation of ROS) not only inhibits the activity of various enzymes but also induces oxidative damage to cellular components such as DNA, protein, and lipids [15,16]. Concurrently, ROS affect cellular function and modulate stress-related primary and secondary metabolites and disturb redox homeostasis [9]. Moreover, ROS cause chlorophyll degradation and reduction of membrane stability [4,14]. A prolonged water deficit may cause cell death as a result of the massive production of ROS, which inhibits the scavenging action of the antioxidants machinery [17]. To prevent oxidative damage, plants have evolved adaptive mechanisms including upregulation of antioxidant defense system activity, which includes ROS-scavenger enzymes (e.g., ascorbate peroxidase (APX), catalase (CAT), glutathione reductase (GR), and superoxide dismutase (SOD)) and non-enzymatic antioxidants (e.g., glutathione, α-tocopherol, ascorbic acid, and phenolic compounds) [18,19,20]. Moreover, the accumulation of osmoprotectants (e.g., glycine betaine, soluble sugars, and proline) contributes to the maintenance of cell turgor by means of osmotic adjustment [4,21]. Therefore, under drought stress, it is imperative to provide sustainable strategies to support plants to resist such stress.

Gibberellins (GA_s_) are phytohormones involved in plant growth and development; stem and root elongation, leaf expansion, flowering, and seed germination, as GA_s_ regulate various metabolic processes, activity of various enzymes, and gene expression [22,23]. Based on previous observations, gibberellic acid (GA_3_) plays a pivotal role in relieving abiotic stress [24,25,26]. Exogenous application of GA_3_ improves stomatal conductance, net photosynthesis rate, ion uptake, and hormonal balance [25]. Besides enhancing water use efficiency (WUE) [22,24], GA_3_ boosts antioxidant capacity [15], minimizes lipid peroxidation, and upregulates enzymatic antioxidants and osmoprotectants [27,28] to mitigate the adverse influences of drought stress. GA_s_ crosstalk with other phytohormones to regulate several metabolic processes during plant growth [29,30]. The biosynthesis of GA_s_ is promoted by indole-3-acetic acid, while GA_s_ catabolize ABA [25,29].

Biostimulants are a promising sustainable strategy to stimulate plant growth and productivity and to strengthen the plant’s ability to mitigate abiotic stresses [19,31,32]. Although the use of commercially available plant growth stimulants such as osmoprotectants and/or antioxidants reduces the deleterious effects of abiotic stress, they are costly to growers. However, natural-based biostimulants such as plant-derived protein hydrolysate, *Moringa oleifera* leaves, propolis, maize grains, licorice roots, and diluted bee honey extracts are inexpensive by-products of plants or organisms that contribute to sustainable agriculture as an alternative to synthetic protectants [26,33,34,35,36,37,38,39]. The direct effect beyond the natural-based biostimulants is due to the fact that they contain many plant growth-promoting molecules such as antioxidants, osmoprotectants, mineral nutrients, and phytohormones. These growth-promoting molecules trigger physiological and biochemical changes, increase water and nutrient uptake, as well as promote resilience against abiotic stress including drought stress [31,36]. Diluted bee honey (Db-H) is a natural solution that mainly contains monosaccharides, disaccharides, and oligosaccharides [40,41]. Moreover, it contains various substances such as minerals, enzymes, proteins, lipids, organic acids, inorganic acids, and phenolic compounds (phenolic acids, flavonoids) [41,42]. Db-H serves as an active antioxidant in scavenging ROS [38,41] due to the presence of flavonoids that inhibit auto-oxidation [42] and enzymes that contribute to the removal of oxygen radicals [41], which are effective protection against drought-induced oxidative damage. As stated by Teklić et al. [32], Bulgari et al. [43], and Semida et al. [38], diluted honey extract as a plant biostimulator can increase tolerance to abiotic stress in plants. A recent field study highlighted the ability of Db-H-based plant biostimulants to alleviate salt stress in onions [38]. Indeed, Db-H applied to onion leaves showed higher biomass production, bulb yield, WUE, and leaf photosynthetic pigment contents. Moreover, Db-H promoted both enzymatic and non-enzymatic antioxidants, membrane integrity, and water content in onion tissues under the influence of salt stress. 

However, to our knowledge, exogenous applications with Db-H as a natural biostimulant along with GA_3_ to plants grown under drought stress have not been studied before. Therefore, the current study was planned to evaluate the possibility of using some growth regulators; Db-H or GA_3_ as a promising tool to relieve the adverse influences of water deficit stress on *Vicia faba* productivity. This research is designed to examine potential positive changes in physio-biochemical attributes, antioxidant defense system activity, and accumulation of osmoprotectants in faba bean plants growing under the influence of drought stress and foliar application of Db-H or GA_3_. In this research, the potential improvement in plant growth, yield, WUE, and photosynthetic efficiency mediated by exogenous application of Db-H or GA_3_ under drought stress conditions was also evaluated.

## 2. Results

### 2.1. Growth and Green Pod Yield

The results in Table 1 show that drought stress significantly decreased the growth traits of *Vicia faba* plants (leaf area plant^−1^, the number of leaves plant^−1^, and shoot dry weight plant^−1^) by 22% and 23%, 26%, and 25%, and 41% and 43% in the 2018/2019 and 2019/2020 seasons, respectively, compared to the control. However, exogenously-applied GA_3_ or Db-H notably increased all growth traits (Db-H recorded better enhancements) compared to the corresponding control. Foliar application of GA3 or Db-H to drought-stressed plants resulted in positive effects on faba bean growth characteristics and recorded identical values for plants grown under full irrigation without the use of any growth regulator (100% of ETc). These effects of water deficit and foliar application of growth regulators on growth traits are reflected on the yield component. Irrigation of faba bean plants with 60% of ETc markedly decreased the green pods’ number plant^−1^ by 30% and 29% and green pods’ yield by 48% and 45% in both seasons, respectively, compared to the control (100% of ETc). However, exogenously-applied GA_3_ or Db-H to faba bean plants compensated the yield reduction occurred through inducing substantial increases in the number of green pods per plant by 65% and 66% and green pods yield by 134 and 138% (seasons average) in the plants subjected to 60% of ETc, respectively, when compared with the corresponding control. It can be seen that the corrective action of GA_3_ and Db-H can bring the pods yield achieved under drought stress to the same yield as achieved under optimum irrigation (100% of ETc). Under the tested irrigation regimes, WUE was differed, meaning that full irrigation recorded a WUE increase of 14% and 8% in both seasons, respectively, compared to the treatment of water deficit. The highest WUE corresponded with 100% of ETc × Db-H treatment, while the 60% of ETc × control treatment recorded the lowest WUE. However, foliar-applied GA_3_ and Db-H to drought-stressed faba bean plants increased WUE by 63% and 66% (seasons average), respectively, compared to those obtained under fully irrigated plants that were not treated with any of the growth regulators (Table 1).

### 2.2. Efficiency of the Photosynthetic Machinery

As displayed in Table 2, water deficit (660% of ETc) caused a considerable decrease in the leaf photosynthetic pigments (total chlorophylls and carotenoids contents), photochemical activity, SPAD chlorophyll index (soil–plant analysis development) values, and photosynthetic efficiency (F_v_/F_m_ and performance index; PI) compared to full irrigation (100% of ETc).

Compared to untreated control plants, sprayed plants with GA_3_ or Db-H showed higher photosynthetic pigment contents, SPAD chlorophyll index, photochemical activity, and the efficiency of PSII. In fully irrigated plants, application of GA_3_ or Db-H increased total chlorophylls by 15% and 24%, total carotenoids by 10% and 18%, photochemical activity by 8% and 13%, SPAD index by 8% and 14%, *F_v_/F_m_* by 6% and 11%, and performance index by 7% and 20% (seasons average), respectively, in comparison to the corresponding control. Foliage-applied GA_3_ or Db-H alleviated the negative effects on the photosynthetic machinery in drought-stressed faba bean plants. In deficit-irrigated plants, the increases in the photosynthetic machinery (total chlorophylls, total carotenoids, photochemical activity, SPAD chlorophyll index, F_v_/F_m_, PI) were 59% and 62%, 27% and 31%, 32% and 32%, 40% and 41%, 18% and 21%, and 52% and 53% (seasons average), respectively, compared with the corresponding control.

### 2.3. Leaf Tissue Stability and Oxidative Stress Indicators

Faba bean leaf tissue stability was assayed as the membrane stability index (MSI), electrolyte leakage (EL), and relative water content (RWC) (Table 3). For irrigation levels, the adverse effects of drought-induced stress on *Vicia faba* plants were described as decreases in RWC and MSI by 16% and 20%, while EL increased by 75% (seasons average), respectively, compared to irrigation with 100% of ETc. Regarding the foliar application of growth regulators, application of GA_3_ or Db-H elevated both RWC and MSI, while minimized EL compared to untreated plants (control). However, GA_3_ or Db-H supplementation markedly attenuated the drought-induced damage to tissue stability in faba bean plants, as the same RWC, MSI, and EL values were recorded for well-watered plants that were not treated with any of the growth regulators.

The utility of the oxidative damage indicators identified in this study was lipid peroxidation, expressed in malondialdehyde (MDA) content, hydrogen peroxide (H_2_O_2_), and superoxide (O_2_^•−^) contents (Table 3). For irrigation level, when irrigation level decreased from 100% to 60% of ETc, MDA, H_2_O_2_, and O_2_^•−^ contents increased by 67%, 58%, and 102%, and 67%, 60%, and 100% in both seasons, respectively. Regarding the growth regulator applications, GA_3_ or Db-H significantly decreased levels of MDA, H_2_O_2_, and O_2_^•−^ compared to the control. For integrative treatments under full irrigation, the best treatments were 100% of ETc × GA_3_ or Db-H which significantly decreased the oxidative stress biomarkers. Under water deficit (60% of ETc), the best treatment was 60% of ETc × GA_3_ or Db-H, which significantly reduced MDA, H_2_O_2_, and O_2_^•−^ contents by 64% and 69%, 52% and 54%, and 69% and 69% (seasons average), respectively, compared to the corresponding control (60% of ETc).

### 2.4. Osmoprotectant Compounds

Results of Table 4 display the contents of the osmoprotectants in terms of soluble sugars, free proline, glycine betaine, and total soluble protein, which increased significantly by 43%, 64%, 85%, and 21% (seasons average) in drought-stressed plants. Nevertheless, under different irrigation regimes, the application of GA_3_ or Db-H increased the contents of soluble sugars, free proline, and glycine betaine contents, while the total soluble protein content was decreased. Under optimum irrigation (100% of ETc), the increases were 43% and 74%, 31% and 31%, and 38% and 38% (seasons average), respectively, compared with the respective control. For osmotically-stressed plants sprayed with GA_3_ or Db-H, the elevations in the soluble sugars, free proline, and glycine betaine contents were 13% and 27%, 21% and 23%, and 30% and 32% (seasons average), respectively in comparison to the corresponding control.

### 2.5. Antioxidant Defense System Components

The contents of non-enzymatic antioxidants (glutathione (GSH), ascorbic acid (AsA), and α-tocopherol (α.TOC)) (Table 5), and enzymatic antioxidant activities (superoxide dismutase (SOD), glutathione reductase (GR), catalase (CAT), and ascorbate peroxidase (APX)) (Table 6) were increased by 49%, 74%, 40%, 25%, 55%, 51%, 60%, and 69% (seasons average), respectively, under the irrigation level of 60% of ETc compared to well-watered plants. However, foliar-applied GA_3_ or Db-H substantially elevated the antioxidant capacity, while the total phenolic compounds were decreased. Under full irrigation, exogenously-applied GA_3_ or Db-H increased the activities of AsA (by 27% and 53%), GSH (by 29% and 58%), α.TOC (by 20% and 37%), SOD (by 16% and 15%), CAT (by 28% and 27%), GR (by 26% and 25%), and APX (by 14% and 14%) (seasons average), respectively, compared with the respective control, but not reached the activities obtained under drought stress.

Under water deficit stress, treatment with GA_3_ or Db-H increased these antioxidant activities by 16% and 16%, 21% and 24%, 11% and 12%, 25% and 26%, 15% and 15%, 21% and 20%, and 18% and 18% (seasons average), respectively, in relation to the corresponding control, and markedly exceeded those obtained under full irrigation (100% of ETc) treatment.

### 2.6. Nutrient Contents

In both seasons, faba bean plants exposed to a water deficit showed significant reductions in the contents of N (by 21%), P (by 23%), K (by 19%), Fe (by 20%), Mn (by 20%) and Zn (by 20%) in comparison to fully irrigated plants (Table 7). Regardless of irrigation levels, applying growth regulators (GA_3_ or Db-H), especially Db-H, markedly increased the nutrient contents compared to untreated plants. Foliar-applied GA_3_ or Db-H attenuated the adverse impact of drought on plant nutritional status. Where, 60% of ETc × GA_3_ or Db-H treatment exhibited higher nutrient contents compared with 60% of ETc, recording values similar to or higher than values of full irrigated plants. The greatest nutrient contents were obtained under 100% ETc × Db-H treatment.

### 2.7. Phytohormone Concentrations

The phytohormone analyses (IAA, GA_3_, CKs, and ABA) displayed differences between the two irrigation regimes (Table 8). Drought-stressed plants exhibited lower IAA (by 23%), GA_3_ (by 26%), and CKs (by 25%), and higher ABA (by 50%) (seasons average) than non-stressed plants. As for the application of plant growth regulators, GA_3_- or (Db-H)-treated *Vicia faba* plants showed higher IAA, GA_3,_ and CKs contents, and lower ABA content than untreated plants. 

The combination of these two factors (irrigation regimes and growth regulators) significantly increased IAA, GA_3,_ and CK_S_ contents, while decreased ABA content (Table 8). Interactive application of GA_3_ or Db-H + full irrigation (100% of ETc) increased IAA (by 20% and 55%), GA_3_ (by 118%% and 39%), and CKs (by 36% and 68%) (seasons average) compared to the respective control. Similarly, foliar-applied GA_3_ or Db-H to plants subjected to water deficit (60% of ETc) notably increased IAA (by 45% and 74%), GA_3_ (by 172% and 59%), and CKs (by 48% and 98%), while decreased ABA (by 49% and 59%) (seasons average) compared to the corresponding control.

## 3. Discussion

In dry regions including Egypt, drought stress is the major constraint to most crop plants, seriously limiting plant growth and productivity and regulating metabolism through complex and various mechanisms linked to plant metabolic pathways [4,12]. Under constant water deficit, plants are unable to withstand such stress through the available endogenous antioxidant defense system as in the case of the *Vicia faba* plants used in the current research. Therefore, *Vicia faba* plants must be supported by exogenous plant growth regulators that may stimulate several physio-biochemical processes, increase plant performance, and enhance resilience against water deficit stress. As presented in Table 10, Db-H analysis showed that this promising tool for sustainable cultivation is a plant growth biostimulator for drought-stressed bean plants. Db-H is rich in osmoprotectants (i.e., proline, total amino acids, and soluble sugars), different sugars, and mineral nutrients (i.e., K, P, Mg, Ca, S, Fe, Mn, Zn, Cu, I, Na, and Se). Additionally, it has high values of vitamins (vitamin C and B-group vitamins). Moreover, Db-H possesses a high value of DPPH radical-scavenging activity (88.2%), which is widely used for screening the antioxidant activity to prevent lipid peroxidation [17,38], which confers the antioxidant property of Db-H. Moreover, exogenously-applied GA_3_ has been reported to induce various metabolic reactions to ameliorate abiotic stress [27,44]. Therefore, as shown in the current study, both GA_3_ and Db-H have crucial mechanisms in favor of drought-stressed *Vicia faba* plants to boost their tolerance to drought stress.

In this study, lowering the irrigation level from 100% to 60% ETc restricted faba bean performance (growth and productivity; Table 1), impaired efficiency of photosynthesis machinery (Table 2), and disrupted leaf tissue stability (RWC and MSI; Table 3). As a result, lipid oxidation (MDA) was increased as a result of the excessive generation of oxidative stress markers (H_2_O_2_ and O_2_^•‒^) (Table 3), associated with increased osmoprotectant compounds (Table 4), and upregulation of non-enzymatic (Table 5) and enzymatic antioxidants (Table 6), which cope with oxidative damage under drought stress [20]. Adverse effects exacerbated by water deficit may be ascribed to osmotic stress with loss of cell turgor and/or ROS overproduction under drought stress [11,23,45]. Nonetheless, foliar-applied GA_3_ or Db-H ameliorated the adverse impacts caused by drought stress on the growth of faba bean plants, thus enhancing green pods yields to be comparable to those of well-watered plants that had not been treated with growth regulators, thus increasing WUE. Under irrigation with 100% of ETc, the improvement in growth and yield of bean plants was more evident by Db-H foliar spray resulting in higher WUE. The recovery of growth and productivity of drought-stressed *Vicia faba* plants by application of GA_3_ or Db-H revealed that these growth regulators may include mechanisms to mitigate the effects of drought-induced stress. This is likely attributed to the growth-related metabolites of Db-H dissolved substances such as proline, soluble sugars, amino acids, antioxidants, vitamins, and mineral nutrients, which support plants to restore their growth and development under drought stress [46,47]. Furthermore, GA_3_ upregulates the expression of genes (xyloglucan endotransglycosylases, expansins, and cyclin-dependent protein kinases) involved in increased cell division and elongation [48]. Moreover, GA_3_ induces osmoregulation by maintaining the osmotic potential, promoting enzyme activity, improving membrane permeability to facilitate mineral nutrient uptake and photosynthesis transportation [22,49,50], thus stimulating plant growth and biomass production (Table 1).

RWC is a physiological indicator of available water content in favor of tissue metabolism, while the degree of membrane integrity can be assessed as MSI and EL [51,52]. Both growth regulators (Db-H and GA_3_) mediated recovery of stressed leaf tissues by increasing cell turgor (RWC) and membrane integrity (MSI), while ion leakage (EL) was reduced (Table 3). The improvement in RWC of drought-stressed plant tissues and cells helped maintain cell turgor through the accumulation of osmolytes such as proline, soluble sugars, and glycine betaine (Table 4) due to Db-H and GA_3_ application and/or changes in elasticity of the cell wall [9,53]. This allowed for continued metabolic activities as effective mechanisms for drought tolerance in stressed faba bean plants. RWC enhanced by exogenous application of Db-H or GA_3_ was closely related to increased WUE in faba bean plants. (Table 1). In this study, the increased protective compounds such as osmoprotectants, enzymatic antioxidants, and low molecular-weight antioxidants (Table 4, Table 5 and Table 6) by foliar-supplemented Db-H or GA_3_ protected plasma membranes from lipid peroxidation (in term of MDA) by decreasing H_2_O_2_ and O_2_^•‒^ contents (Table 3). These findings may be related to improved MSI, decreased EL and photo-oxidation, and enhanced membrane integrity against oxidative damage [38,46], and thus improved faba bean plant growth and outputs under water deficit stress. 

In the current study, leaf photosynthetic pigment contents (total chlorophylls and carotenoids), photochemical activity, SPAD chlorophyll index, and photosynthetic efficiency (F_v_/F_m_ and PI) were reduced while the irrigation water was reduced to 60% ETc, indicating chlorophyll degradation in chloroplasts and photoinhibition of PSII of water-stressed faba bean plants due to the damaging influences of ROS [54,55]. However, leaf photosynthetic pigment contents, photochemical activity, SPAD chlorophyll index, and photosynthetic efficiency (Table 2) were markedly improved by foliar-applied Db-H [38] or GA_3_ [22]. These results may be related to maintaining cell membrane integrity and increasing leaf RWC by Db-H or GA_3_ supplementation. Both Db-H and GA_3_ likely mitigated the negative effects of drought, and faba bean plants responded to drought stress by up-regulation of osmoprotectants (Table 4), non-enzymatic (Table 5) and enzymatic antioxidants (Table 6) for ROS-scavenging to minimize lipid peroxidation. In line with our findings, GA_3_ supplementation improved leaf chlorophyll content in wheat [27] and maintained the photosynthetic efficiency of PSII in laurel seedlings [56]. Additionally, Db-H is rich in nutrients to maintain intercellular hemostasis of ions required for photosynthetic biosynthesis, thus improving the efficiency of the photosynthetic machinery of *Vicia faba* plants. 

Nutrients deficiency in plants that is attributed to the osmotic impact of water deficit stress and/or soil water deficit disturbs nutrient availability, uptake, translocation, and metabolism [9], which lead to the reduction of macro-and micro-nutrients contents in drought-stressed faba bean (Table 7). Nevertheless, foliar-applied GA_3_ or Db-H induced ion hemostasis and increased mineral nutrient contents of drought-stressed plants. This may be attributed to that exogenous application of GA_3_ or Db-H increased root uptake surfaces resulting from increased root system volume (data not shown), and/or increased accumulation of osmoprotectants (Table 4) to balance the osmotic pressure in organelles, thus mainlining cell turgor and improving nutritional status and water uptake [57]. 

In this work, the plant defense machinery including synthesis of osmoprotectants (proline, soluble sugars, glycine betaine, and total soluble protein; Table 4), and both non-enzymatic antioxidants contents (AsA, GSH, and α.TOC; Table 7), and enzymatic antioxidants activities (SOD, CAT, GR, and APX; Table 6) increased in growth regulators (GA_3_ and Db-H)-treated plants. This positive situation protected faba bean plants from the deleterious impacts of water deficit stress by osmotic adjustment and ROS-scavenging [15,38]. Increased osmoprotectants are likely to lead to the uptake or breakdown of Db-H as biostimulants, given that it is rich in osmoprotectant compounds (Table 9). Furthermore, GA_3_ regulates different genes that can modulate the osmotic ability to maintain cell enlargement through the accumulation of osmotically active solutes such as soluble sugar, soluble protein, free proline, and glycine betaine [28,58]. Our results showed that drought stress in combination with either of the growth regulators (GA_3_ or Db-H) markedly improved the antioxidant defense system to enable *Vicia faba* plants to withstand drought stress through protection from oxidative damage as evidenced by the decreased contents of MDA, H_2_O_2_, and O_2_^•‒^ (Table 3).

It has been well demonstrated that phytohormones play an important role in various physiological, biochemical, and molecular processes in plants to mitigate drought stress [59], which was significantly increased by exogenous application of GA_3_ or Db-H, while ABA content was reduced (Table 8). In this study, Db-H promoted the contents of IAA, CKs, and GA_3_ in faba bean plants subjected to drought stress (Table 8), which could be attributed to the increased mineral nutrients required for the formation of protoplasm and phytohormones [38]. According to Semida and Rady [34], presoaking bean seeds with some extracts resulted in higher contents of IAA and GA_3_, while decreased ABA. Different genes are expressed after GAs treatment highlighting that GAs upregulated genes related to IAA and other genes related to ABA are down-regulated by GAs [58], while CKs have antagonistic roles against ABA [34]. Further, GAs-induced degradation of DELLA proteins is modulated by different signals such as salinity and drought, and other hormones [60], revealing that GAs regulate and crosstalk with other phytohormones to ameliorate the deleterious effects of drought stress. Water deficit stress disrupts the hormonal balance in plants, and thus, hormonal hemostasis may be a means for GA_3_-induced drought stress tolerance [25].

Finally, the negative effects of environmental foes may exceed the natural endurance of stressed plants. In this case, the components of a stressed plant’s defense system do not meet adequate defense requirements, and therefore external use of auxiliary substances such as nutrients and other beneficial strategies increases the efficiency of antioxidant defenses, and thus plants can perform efficiently under adverse conditions of environmental foes [61,62,63,64,65].

## 4. Materials and Methods

### 4.1. Experimental Location and Soil Properties

Using a private farm (Fayoum; 29.3452 N, 30.5686 E, Egypt), two experiments were conducted at the field level during two consecutive winter seasons (2019 and 2020). The soil, 0.90–1.0 m deep, with loamy sand texture, which is classified as Typic Torripsamments, siliceous, hypothermic [66]. The soil physical and chemical properties were performed applying methods described in Klute [67] and Page et al. [68], and results are shown in Table 10. The electrical conductivity of the tested soil was 8.23 dS m^−1^, being saline soil according to the classification of Dahnke and Whitney [69].

### 4.2. Planting, Treatments, and Experimental Layout

The seeds of *Vicia faba* (cv. Giza 40; widespread cultivar of faba bean in the study area based on the recommendation of the Egyptian Ministry of Agriculture) were secured from the Agricultural Research Center, Egypt. Firstly, the seeds were washed with distilled water then sterilized with sodium hypochlorite solution (1%; *v/v*) for roughly two min, once more the seed surface was cleaned from sterilization solution with distilled water after that were kept at room temperature to dry. The seeds were sown on October 20, for both seasons (2019 and 2020) in hills with plant and row spacing of 25×70 cm. Each plot area was 10.5 m^2^; 3.5 m length (5 rows) × 3 m width. 

In this study, there are two treatment factors; including irrigation regimes and exogenous application of plant growth regulators. Two irrigation regimes were applied corresponding with 100% and 60% of the crop evapotranspiration (ETc). Gibberellic acid (GA_3_) and diluted bee honey (Db-H) were applied at 20 mg and 20 g L^−1^, respectively, as foliar spraying. These concentrations were selected based on our preliminary pot study (Table S1). The irrigation treatments were separated by a 1 m non-irrigated area. Until the full emergence of seedlings (15 days after planting; DAP), the faba bean plants were irrigated at 100% of ETc to ensure good plant establishment, thereafter the two irrigation treatments were initiated. These two irrigation treatments were chosen based on our preliminary pot study (Table S1). Fifteen days after the initiation of the irrigation treatments, GA_3_ and Db-H were applied as foliar spraying in the early morning. Fifteen days after the first spraying, the second foliar spray was implemented for faba bean plants. Sprays were conducted to run-off, with the use of Tween-20 (0.1%, v/v) as a surfactant to ensure optimum penetration into leaf tissues. The plants (*n* = 200) in each experimental unit (10.5 m^2^) were sprayed with 2 L of spray solution, which was increased to 2.4 L for the second time of spraying. The experimental layout for each treatment was designed as a Randomized Split Plot with three replications. Different fertilizers (5 tons organic manure, 50 kg potassium humate, 75 kg of P_2_O_5_ using Ca(H_2_PO_4_)_2_; 15.5% P_2_O_5_, 60 kg of K_2_O using K_2_SO_4_; 48% K_2_O, and 45 kg of N using (NH_4_)_2_SO_4_; 21% N were added per hectare) and agronomic practices were applied following the recommendations of the Agricultural Research Center, Giza, Egypt.

### 4.3. Irrigation Water Applied (IWA) 

The reference evapotranspiration (ETo) was given using the class A pan data (E_pan_; mm day^−1^), adjacent to the experimental plots adjusted with appropriate pan coefficient (K_pan_) and the crop coefficient (K_c_) [70]. The ETc (mm day^−1^) was determined as the following formula [70]:

ETc = E_pan_ × K_pan_ × K_c_ (1)

Irrigation water applied (IWA) was computed with an equation as follows:

IWA = (A × ETc × Ii × Kr) / [Ea × 1000 × (1 − LR)](2)

where, IWA = irrigation water applied (m^3^), A = area of plots (m^2^), ETc = crop water requirements (mm per day), Ii = intervals of irrigation (day), Kr = covering factor, Ea = efficiency of application (%), and LR = requirements for leaching.

The total irrigation water applied during both winter seasons was 3690 and 2214 m^3^ ha^−1^ in the 2019 season and 3754 and 2252 m^3^ ha^−1^ in the 2020 season for 100 and 60% of ETc, respectively. The digital moisture meter sensors (HH2 type, Cambridge, CB5 0 EJ, UK) were utilized to record the water content of the tested soil every two days at different depths, 0–15 and 15–30 cm.

### 4.4. Bee Honey Analysis for Physico-Chemical Composition

Clover honey used in the current study was analysed for effective components and results are shown in Table 10. Moisture (%), proline, and pH were assessed according to AOAC [71]. Quantities of sugars by High-Performance Liquid Chromatography (HPLC) were measured as the concentration of fructose, glucose, maltose and sucrose (%) according to Bogdanov and Baumann [72]. Mineral nutrients were measured according to the methodology given in [73]. Ascorbic acid concentration was determined according to Mukherjee and Choudhuri [74]. Determination of the antioxidant activity was performed using 1,1-diphenyl-2-picrylhydrazyl (DPPH) assay as described by Lee et al. [75].

### 4.5. Sampling and Measurements

#### 4.5.1. Growth and Yield Characteristics, and WUE

Plant growth characteristics were analyzed at sixty DAP in each season, 5 plants were selected, randomly, from each plot (main and sub-main plots). The number of branches and leaves for each plant was counted. The leaf area (cm^2^) was measured using a held-hand planimeter (Planix 7, Tamaya Technics Inc. Tokyo, Japan). The shoot was weighed for each plant to determine to shoot fresh weight (g). For recording shoot dry weight (g), shoots were oven-dried at 70 ± 2 °C until a constant weight was reached. 

On the same date (60 DAP), green pods yield parameters were recorded in terms of the number of green pods per plant and green pods’ weight (ton) per hectare. These parameters of green pods yield were measured using the two outer rows of each experimental plot. The WUE was calculated as presented by Jensen [76]:

WUE = [Green pods yield (kg m^−2^)]/[irrigation water applied (m^−3^ m^−2^)] (3)

#### 4.5.2. Efficiency of the Photosynthetic Machinery

Leaf photosynthetic pigment contents were determined in terms of chlorophylls and carotenoids based on the Arnon [77] procedures. Homogenization in 80% acetone (v/v) and centrifugation at 10,000× *g* for 10 min were implemented. The acetone extract solution absorption was recorded at 663, 645, and 470 nm in a UV–Visible spectrophotometer (UV-160A, Shimadzu, Japan).

Photochemical activity in fresh ear leaf was determined using the Ferricyanide technique as depicted by Jagendorf [78] with some modifications given in the Avron [79] method.

Using the SPAD-502 chlorophyll meter (Minolta, Osaka, Japan), the relative chlorophyll content (soil–plant analysis development (SPAD index) values) was measured. The measurements of chlorophyll “a” fluorescence were performed using a handy PEA chlorophyll fluorometer (Hansatech Instruments Ltd., Kings Lynn, UK). The maximum quantum yield of PSII (F_v_/F_m_) was determined using the equation: F_v_/F_m_ = (F_m_ ‒ F_0_)/F_m_ [80]. The photosynthesis performance index (PI) that quantifies multi-parameters as electron flow rate, absorption, trapping, and dissipation of excitation energy, was computed as described by Clark et al. [81].

#### 4.5.3. Leaf Tissue Stability and Oxidative Stress Biomarkers

Using the fully enlarged upper leaves, the Osman and Rady [82] procedure was practiced to assess the leaf relative water content (RWC). Midribs were excluded and the leaf blades were divided into 2 cm-diameter discs, which were immediately weighed (fresh mass). The discs were then saturated by deionized water for 24 h in the dark, gently surface-dried from the adhering water drops to record the turgid mass. To record dry mass, discs drying was implemented for 48 h under 70 °C, and the following equation was utilized for calculating RWC percentage:

RWC (%) = [(fresh mass − dry mass)/(turgid mass − dry mass)] × 100(4)

Using the fully enlarged upper leaves, midribs were excluded and the leaf blades were divided into 0.2 g leaf pieces to evaluate leaf membrane stability index (MSI) [83]. A sample (0.2 g) was immersed in 10 ml of ion-free water and 40 °C for 30 min was practiced to record EC_1_. Another 0.2 g sample was boiled for 10 min to record EC_2_. The following equation was utilized for calculating MSI percentage:

MSI (%) = [1 − (EC1/EC2)] × 100(5)

Using fully enlarged upper leaves, midribs were excluded and the leaf blades were divided into discs to assess ions leaked from leaf tissue [83]. Using 20 discs immersed in 10 ml of ion-free water, EC_0_ was recorded. EC_1_ was then measured after heating the tube content at 45–55 °C for 30 min. Then, the content of the tube was boiled for 10 min to record EC_2_. The following equation was utilized for calculating electrolyte leakage (EL) percentage:

EL (%) = [(EC2 − EC1)/EC3] × 100(6)

Determination of lipid peroxidation that assessed as malondialdehyde (MDA), and the two biomarkers of oxidative stress; superoxide (O_2_^•‒^), and hydrogen peroxide (H_2_O_2_) contents were implemented applying the procedures of Madhava Rao and Sresty [84], Velikova et al. [85], and Kubiś [86], respectively. The contents of MDA were assessed applying an extinction coefficient (155 mM^−1^ cm^−1^) and presented as µmol g^−1^ FW. The H_2_O_2_ content (µmol g^−1^ FW) was evaluated colorimetrically at 390 nm and the calculations were performed based on a proper standard curve. The O_2_^•−^ content (µmol g^−1^ FW) was evaluated using sample fragments (1 × 1 mm, 0.1 g) that flooded using a buffer (K-phosphate, 10 mM, pH 7.8), which was mixed with each of NBT (0.05%) and NaN_3_ (10 mM) for 60 min under 25 °C. The mixture was subjected to 85 °C for 15 min. The mixture was then cooled rapidly. The absorbance readings were taken at 580 nm.

#### 4.5.4. Contents of Osmoprotectant Compounds

Using toluene, extraction of proline was practiced and at 520 nm, the absorbance was recorded [87]. Leaf content (μg proline g^−1^ FW) of proline was calculated using a suitable standard curve. Glycine betaine (GB) content was estimated under acidic conditions through monitoring formed periodide crystals colorimetrically (at 365 nm) after reaction of the mixture with a reagent (cold KI‒I_2_) [88]. By utilizing a professional method [89], extraction (with 96% ethyl alcohol), and determination of the content of total soluble sugars (mg g^−1^ DW). The reaction of the ethanolic extract (100 µL) was implemented with 150 mg of anthrone as a reagent prepared, freshly, in 100 mL H_2_SO_4_, 72%. Then, the mixture was boiled for 10 min and readings were taken at 625 nm after cooling. The procedures described in Bradford [90] were used to determine total soluble protein content.

#### 4.5.5. Contents of Non-Enzymatic Antioxidant Compounds

Ascorbate (AsA) was determined in the tissue of the upper fully-expanded leaf after the homogenization in HPO_3_ (ice-cold, 5%) contained 1 mM EDTA. The produced homogenates were centrifuged at 4,000 × g for 20 min, and supernatants were used to estimate AsA [91]. Determination of glutathione (GSH) was performed [92] with a minor modification [93] and a known concentration of GSH was used as a standard curve. α-Tocopherol (α-TOC) was detected according to the method of Ching and Mohamed [94] and Konings et al. [95]. The total leaf content of phenolic compounds was assessed by the Folin–Ciocalteu method [96] functioning gallic acid as a standard. At 725 nm, the absorbance readings were recorded and the total phenolic contents were presented as mg gallic acid equivalents (GAE) g^−1^ dry weight, computed from a standard curve prepared with gallic acid.

#### 4.5.6. Activities of Antioxidant Enzymes

The fully enlarged upper leaves were used to extract enzymes in 0.5 g. An ice-cold buffer, pH 7.0 (e.g., 100 mM K-phosphate, which contained 1% PVP) was utilized with a pre-chilled (cleaned) mortar and pestle to macerate leaf samples. The obtained homogenates were transferred for the centrifugation process at 12,000× *g* for 0.25 h under 4 °C. The obtained supernatants were the enzymatic extracts, which were utilized for assaying the activities of catalase (CAT), glutathione reductase (GR), and ascorbate peroxidase (APX). Using the method detailed in Aebi [97], assaying of the CAT activity (Unit mg^−1^ protein) was performed using a spectrophotometer apparatus at 240 nm. To assay the ability of the enzyme to decompose the H_2_O_2_ for 2 min, 2 mL of the reaction mixture of a P-buffer (50 mM, pH 6.0), EDTA (0.1 mM), H_2_O_2_ (0.02 M), and 0.1 mL of the enzymatic extract was applied, and an extinction coefficient (39.4 mM^−1^ cm^−1^) was also applied. The Nakano and Asada [98] method was applied to assay the APX activity (Unit mg^−1^ protein). Using spectrophotometer, 2 mL mixture (P-buffer (50 mM, pH 7.5), EDTA (100 µM), AsA (300 µM), 0.1 mL H_2_O_2_, and 0.1 mL enzyme extract) was observed for 2 min at 290 nm, and 2.8 mM^−1^ cm^−1^ was applied as an extinction coefficient. The Foster and Hess [99] method was applied to assess the GR activity (Unit mg^−1^ protein) by monitoring (for 3 min at 340 nm) the changes that occurred in the reading of the reaction mixture (K-phosphate buffer (0.1 M, pH 7.0), EDTA (100 µM), NADPH (0.5 mM), GSSG (0.1 mM), and 0.1 mL enzyme extract).

Homogenization with ice was performed for frozen samples (500 mg) and the homogenization solution was 10 mL of 50 mM L^−1^ HEPES buffer and 0.l mM L^−1^ Na_2_EDTA (pH 7.6). To obtain a crude extract, centrifugation was practiced for homogenates for a quarter of an hour at 15,000× *g* under 4 °C, which was functioned for assaying protein and superoxide dismutase (SOD). Overnight, dialyzing of crude extract was performed against a diluted homogenizing solution to eradicate the interference in SOD assay from substances having low molecular weights. The protein-dye binding method [90] was functioned to assess the concentration of soluble protein against a standard (bovine serum albumin). Assaying the SOD (EC 1.15.1.1) activity was implemented through inhibiting NBT photochemical reduction under practicing the Yu and Rengel [100] method.

#### 4.5.7. Contents of Nutrient Elements

Digestion process was performed for the dried leaf samples with a mixture consisting of perchloric and nitric acids (at 1: 3, v/v, respectively). Using the previous digestion solution, assessments of N, P, and K^+^ contents were performed. Determination of N was performed using the micro-Kjeldahl apparatus (Ningbo Medical Instruments Co., Ningbo, China) following [101]. The P content was assessed following the blue color method [102] whereby molybdenum was used to reduce molybdophosphoric in sulfuric acid while reducing to exclude arsenic. The K^+^ content was assessed utilizing a flame photometer (Perkin-Elmer Model 52-A, Glenbrook, Stamford, CT, USA) device as depicted in the methods of Page et al. [68]. Micronutrients (Zn, Mn, and Fe) contents were detected in dried leaf samples according to Johnson and Ulrich [103] with atomic absorption spectroscopy under checking against standard reference samples (NIST, USA). 

#### 4.5.8. Contents of Plant Hormones

The phytohormones; indole-3-acetic acid (IAA), gibberellic acid (GA_3_), cytokinins (CKs) profiling were implemented based on the procedures of gas chromatography-mass spectrometry (GC-MS) methods improved by Nehela et al. [104] with minor adjustments [26]. Fresh leaves (0.1 g) sample was extracted in ice-cold extraction solvent (2 mL; methanol/water/HCl (6N); 80/19.9/0.1; *v/v/v*). Then, the extract was centrifuged at 25,000× g, 4 °C for 5 min. Supernatants were collected and concentrated to 50 μL under N stream and then stored at −80 °C until analysis. For IAA, 50 μL of the supernatant was derivatized with 40 μL of MCF then concentrated to 20 μL under N stream and 0.5 mg of Na_2_SO_4_ were added to dry the organic phase. For CKs and GA_3_, 50 μL from the supernatant was dried and derivatized with 100 μL of N-Methyl-N-(trimethylsilyl) trifluoroacetamide (MSTFA) by heating at 85 °C for 45 min. For GC–MS analysis, 1 μL was injected into the GC–MS running in the selective ion mode (SIM-mode). All samples and phytohormone standards were analysed using a Clarus 680 GC with SQ8-T Mass Spectrometer system (Perkin Elmer, Waltham, MA, USA) fitted with an Elite-5MS capillary column (low bleed, 30 m × 0.25 mm × 0.025 μm film thickness; Perkin Elmer, Waltham, MA, USA). Helium was the carrier gas with a flow rate of 1 mL min^−1^. The temperature program for IAA was as the following: the column was held at 50 °C for 3 min, and then increased to 200 °C at a rate of 4 °C min^−1^, held for 5 min. While, the program for CKs and GA_3_ was as the following: the column was held at 60 °C for 2 min and then increased to 160 °C at 20 °C min^−1^, and finally to 290 °C at 5 °C min^−1^. The injector and the detector temperatures were set at 250 °C and 260 °C, respectively. The TurboMass software version 6.1 (Perkin Elmer, Waltham, MA, USA) was used to analyze chromatograms. Identification of IAA, CKs and GA_3_ was performed by comparing their retention time, linear retention indices (LRIs) and the selected ions with those of authentic standards. Extraction and estimation of the content of abscisic acid (ABA) were implemented using high-performance liquid chromatography (HPLC) as outlined by Ünyayar et al. [105].

### 4.6. Statistical Tests

The data were analyzed based on the GLM procedures of the GENSTAT software (VSN International Ltd, Oxford, UK). All data were subjected to the combined analysis and the mean differences were compared with the least significant difference (LSD) test at 5% probability (*p* ≤ 0.05) level. The analyzed results are presented as the mean ± standard error.

## 5. Conclusions

The current study exhibits differences in physiological, biochemical, and metabolic responses among the (Db-H)- or GA_3_-treated and untreated faba bean plants. Exogenous application of Db-H or GA_3_ markedly elevated the level of non-enzymatic and enzymatic antioxidants and osmoprotectants (proline, glycine betaine, soluble sugars, and soluble protein) as well as increased the phytohormones (indole-3-acetic acid and gibberellic acid and cytokinins), this associated with the reduction of malondialdehyde (MDA) and abscisic acid (ABA). Foliar applied Db-H or GA_3_ improved the nutrients status, tissue health, leaf photosynthetic pigments, and photosynthetic efficiency leading to higher growth and productivity (yield and water use efficiency) of drought-stressed faba bean plants. Therefore, the application of these growth regulators (Db-H and GA_3_) was identified to be an effective strategy to mitigate the damage effects of irrigation water deficits for sustainable faba bean production in arid and semi-arid areas.

## Figures and Tables

**Table 1:** Foliar application of some growth regulators (e.g., gibberellic acid; GA_3_ and diluted bee honey; Db-H) promoted growth and green pod yield components of *Vicia faba* plants grown under well watering (100% of crop evapotranspiration; ETc) or deficit irrigation (60% of ETc).

Source of Variation	No. of Leaves per Plant	Leaf Area per Plant (cm^2^)	Shoot DW per Plant (g)	No. of Green Pods per Plant	Green Pods Yield per Hectare (ton)	WUE (Kg per m^3^)
	Season of 2018/2019					
Irrigation (Ir)	*	*	**	*	**	*
100% of ETc	31.8 ± 3.1a	136.0 ± 13.7a	17.7 ± 1.7a	20.2 ± 1.7a	32.5 ± 2.9a	8.81 ± 1.12a
60% of ETc	24.8 ± 2.4b	100.7 ± 10.1b	10.4 ± 1.0b	14.2 ± 1.3b	16.8 ± 1.7b	7.59 ± 1.09b
Regulators (Re)	*	*	*	*	*	*
Control (Cn)	23.5 ± 2.3c	95.0 ± 10.3c	10.1 ± 1.0c	13.3 ± 1.3c	15.1 ± 1.5c	5.12 ± 0.88c
GA_3_	29.7 ± 3.0b	125.8 ± 12.6b	15.0 ± 1.6b	18.2 ± 1.4b	26.9 ± 2.3b	9.11 ± 1.03b
Db-H	31.7 ± 3.0a	134.3 ± 12.9a	17.2 ± 1.6a	20.0 ± 1.9a	32.1 ± 3.1a	10.87 ± 1.21a
Ir × Re	*	*	*	*	*	*
100% ETc × Cn	28.3 ± 2.7c	117.5 ± 12.1c	11.9 ± 1.2c	16.5 ± 1.3c	20.8 ± 1.8c	5.64 ± 0.98d
100% ETc × GA_3_	31.7 ± 3.4b	137.7 ± 14.2b	18.5 ± 1.9b	20.3 ± 1.5b	33.3 ± 2.4b	9.02 ± 1.13b
100% ETc × Db-H	35.3 ± 3.2a	152.7 ± 14.8a	22.7 ± 2.1a	23.7 ± 2.4a	43.5 ± 4.4a	11.79 ± 1.23a
60% ETc × Cn	18.7 ± 1.9c	72.4 ± 8.4d	8.2 ± 0.7d	10.1 ± 1.3d	9.4 ± 1.1d	4.25 ± 0.86c
60% ETc × GA_3_	27.7 ± 2.6c	113.8 ± 11.0c	11.4 ± 1.2c	16.1 ± 1.3c	20.4 ± 2.1c	9.21 ± 0.99b
60% ETc × Db-H	28.0 ± 2.8c	115.9 ± 10.9c	11.7 ± 1.0c	16.3 ± 1.4c	20.7 ± 1.8c	9.35 ± 1.21b
	**Season of 2019/2020**					
Irrigation (Ir)	*	*	**	*	**	*
100% of ETc	33.1 ± 3.2a	152.4 ± 13.3a	19.8 ± 2.0a	19.1 ± 2.2a	31.2 ± 3.1a	8.31 ± 0.93a
60% of ETc	25.6 ± 2.4b	113.9 ± 11.1b	11.2 ± 1.0b	13.5 ± 1.5b	17.2 ± 1.7b	7.64 ± 1.02b
Regulators (Re)	*	*	*	*	*	*
Control (Cn)	24.2 ± 2.4b	105.6 ± 9.3c	10.7 ± 1.1c	12.6 ± 1.4c	15.2 ± 1.7c	5.06 ± 1.03c
GA_3_	31.0 ± 3.0a	140.8 ± 12.3b	17.2 ± 1.8b	17.3 ± 2.0b	26.6 ± 2.6b	8.86 ± 1.16b
Db-H	33.0 ± 3.2a	151.4 ± 15.1a	18.7 ± 1.7a	19.2 ± 2.2a	30.9 ± 3.1a	10.29 ± 1.32a
Ir × Re	*	*	*	*	*	*
100% ETc × Cn	29.4 ± 2.8c	134.1 ± 10.4c	12.7 ± 1.3c	15.9 ± 1.8c	21.8 ± 2.3c	5.81 ± 0.69d
100% ETc × GA_3_	33.1 ± 3.2b	150.2 ± 12.2b	21.9 ± 2.2b	18.8 ± 2.2b	31.7 ± 2.8b	8.44 ± 1.06c
100% ETc × Db-H	36.7 ± 3.7a	169.4 ± 17.4a	24.8 ± 2.3a	22.7 ± 2.5a	40.1 ± 4.2a	10.68 ± 1.22a
60% ETc × Cn	18.9 ± 1.9d	77.1 ± 8.2d	8.7 ± 0.9d	9.2 ± 1.0d	8.5 ± 1.0d	3.77 ± 0.63e
60% ETc × GA_3_	28.8 ± 2.7c	131.4 ± 12.4c	12.4 ± 1.2c	15.7 ± 1.7c	21.4 ± 2.3c	9.50 ± 1.11b
60% ETc × Db-H	29.2 ± 2.6c	133.3 ± 12.7c	12.6 ± 1.0c	15.7 ± 1.8c	21.7 ± 1.9c	9.63 ± 1.13b

** and * indicate respectively differences at *p* ≤ 0.05 and *p* ≤ 0.01 probability level. Means followed by the same letter in each column are not significantly different according to the LSD test (*p* ≤ 0.05).

**Table 2:** Foliar application of some growth regulators (e.g., gibberellic acid; GA_3_ and diluted bee honey; Db-H) promoted photosynthetic machinery efficiency of *Vicia faba* plants grown under well watering (100% of crop evapotranspiration; ETc) or deficit irrigation (60% of ETc).

Source of Variation	Total Chlorophylls (mg per g FW)	Total Carotenoids (mg per g FW)	Photochemical Activity	SPAD Chlorophyll Index	*F_v_/F_m_*	Performance Index (%)
	Season of 2018/2019					
Irrigation (Ir)	*	*	*	*	*	*
100% of ETc	3.13 ± 0.19a	0.75 ± 0.02a	45.2 ± 1.6a	66.6 ± 2.4a	0.85 ± 0.02a	16.8 ± 0.21a
60% of ETc	2.44 ± 0.12b	0.63 ± 0.01b	38.9 ± 1.3b	56.8 ± 1.9b	0.77 ± 0.02b	13.8 ± 0.17b
Regulators (Re)	*	*	*	*	*	*
Control (Cn)	2.33 ± 0.12c	0.60 ± 0.01c	37.5 ± 1.2b	54.0 ± 1.8b	0.75 ± 0.02b	13.0 ± 0.18c
GA_3_	2.94 ± 0.19b	0.72 ± 0.02b	43.7 ± 1.6a	64.7 ± 2.5a	0.83 ± 0.03a	16.0 ± 0.19b
Db-H	3.10 ± 0.15a	0.76 ± 0.02a	45.0 ± 1.7a	66.4 ± 2.2c	0.86 ± 0.03a	17.1 ± 0.22a
Ir × Re	*	*	*	*	*	*
100% ETc × Cn	2.78 ± 0.17c	0.68 ± 0.01c	42.3 ± 1.3b	62.4 ± 2.1c	0.81 ± 0.02b	15.8 ± 0.21b
100% ETc× GA_3_	3.18 ± 0.21b	0.76 ± 0.02b	45.4 ± 1.6a	67.1 ± 2.7a	0.85 ± 0.02ab	16.3 ± 0.18b
100% ETc × Db-H	3.42 ± 0.18a	0.82 ± 0.02a	47.9 ± 2.0a	70.2 ± 2.4a	0.89 ± 0.03a	18.4 ± 0.25a
60% ETc × Cn	1.87 ± 0.07d	0.52 ± 0.00d	32.6 ± 1.1c	45.6 ± 1.4d	0.69 ± 0.01c	10.1 ± 0.14c
60% ETc × GA_3_	2.69 ± 0.16c	0.68 ± 0.01c	41.9 ± 1.5b	62.3 ± 2.2c	0.80 ± 0.03b	15.6 ± 0.20b
60% ETc × Db-H	2.77 ± 0.12c	0.70 ± 0.02c	42.1 ± 1.3b	62.5 ± 2.0c	0.82 ± 0.02b	15.7 ± 0.18b
	**Season of 2019/2020**					
Irrigation (Ir)	*	*	*	*	*	*
100% of ETc	3.41 ± 0.14a	0.76 ± 0.03a	46.1 ± 1.6a	68.2 ± 2.2a	0.85 ± 0.03a	17.2 ± 0.17a
60% of ETc	2.60 ± 0.11b	0.65 ± 0.01b	39.2 ± 1.5b	57.1 ± 2.0b	0.75 ± 0.02b	13.7 ± 0.13b
Regulators (Re)	*	*	*	*	*	*
Control (Cn)	2.38 ± 0.10b	0.63 ± 0.01b	37.5 ± 1.5b	53.9 ± 2.1b	0.73 ± 0.02b	12.9 ± 0.14b
GA_3_	3.26 ± 0.13a	0.73 ± 0.02a	44.6 ± 1.8a	65.7 ± 2.0a	0.83 ± 0.02a	16.3 ± 0.16a
Db-H	3.40 ± 0.14a	0.76 ± 0.03a	46.0 ± 1.6a	68.3 ± 2.3a	0.85 ± 0.04a	17.2 ± 0.16a
Ir × Re	*	*	*	*	*	*
100% ETc × Cn	3.01 ± 0.12b	0.70 ± 0.02b	43.1 ± 1.5b	63.5 ± 2.4b	0.80 ± 0.03b	15.5 ± 0.16c
100% ETc× GA_3_	3.49 ± 0.15a	0.76 ± 0.02a	46.4 ± 1.8a	68.1 ± 1.9a	0.86 ± 0.02a	17.1 ± 0.18b
100% ETc × Db-H	3.74 ± 0.14a	0.81 ± 0.04a	48.9 ± 1.6a	72.9 ± 2.2a	0.89 ± 0.04a	19.0 ± 0.18a
60% ETc × Cn	1.74 ± 0.08c	0.56 ± 0.00c	31.8 ± 1.4c	44.3 ± 1.7c	0.66 ± 0.01c	10.3 ± 0.11d
60% ETc × GA_3_	3.02 ± 0.11b	0.69 ± 0.02b	42.8 ± 1.7b	63.3 ± 2.1b	0.79 ± 0.02b	15.4 ± 0.14c
60% ETc × Db-H	3.05 ± 0.14b	0.71 ± 0.02b	43.0 ± 1.5b	63.6 ± 2.3b	0.80 ± 0.04b	15.4 ± 0.14c

** and * indicate respectively differences at *p* ≤ 0.05 and *p* ≤ 0.01 probability level. Means followed by the same letter in each column are not significantly different according to the LSD test (*p* ≤ 0.05).

**Table 3:** Foliar application of some growth regulators (e.g., gibberellic acid; GA_3_ and diluted bee honey; Db-H) promoted leaf tissue stability and levels of oxidative stress biomarkers in *Vicia faba* plants grown under well watering (100% of crop evapotranspiration; ETc) or deficit irrigation (60% of ETc).

Source of Variation	Relative Water Content (%)	Membrane Stability Index (%)	Electrolyte Leakage (%)	Malondialdehyde Level (µmole per g FW)	Hydrogen Peroxide (H_2_O_2_) Level (µmole per g FW)	Superoxide (O_2_^•‒^) Level (µmole per g FW)
	Season of 2018/2019					
Irrigation (Ir)	*	*	**	**	**	**
100% of ETc	87.6 ± 4.6a	76.3 ± 3.8a	10.6 ± 0.5b	0.12 ± 0.01b	1.29 ± 0.03b	0.50 ± 0.01b
60% of ETc	74.0 ± 4.3b	61.2 ± 3.3b	18.9 ± 1.0a	0.20 ± 0.01a	2.04 ± 0.02a	1.01 ± 0.02a
Regulators (Re)	*	*	*	*	*	*
Control (Cn)	70.5 ± 3.8b	55.6 ± 3.3b	22.4 ± 1.3a	0.24 ± 0.02a	2.29 ± 0.05a	1.22 ± 0.03a
GA_3_	85.2 ± 4.7a	75.1 ± 3.8a	11.0 ± 0.6b	0.13 ± 0.01b	1.37 ± 0.02b	0.53 ± 0.02b
Db-H	86.8 ± 4.9a	75.5 ± 3.6a	10.8 ± 0.5b	0.12 ± 0.00b	1.34 ± 0.02b	0.52 ± 0.01b
Ir × Re	*	*	*	*	*	*
100% ETc × Cn	82.6 ± 4.5b	72.8 ± 3.3b	11.1 ± 0.6b	0.13 ± 0.01b	1.46 ± 0.04b	0.55 ± 0.02b
100% ETc × GA_3_	88.9 ± 4.2a	77.9 ± 4.1a	10.4 ± 0.6b	0.12 ± 0.01b	1.21 ± 0.02c	0.48 ± 0.01c
100% ETc × Db-H	91.4 ± 5.1a	78.1 ± 3.9a	10.2 ± 0.4b	0.12 ± 0.00b	1.19 ± 0.02c	0.47 ± 0.01c
60% ETc × Cn	58.3 ± 3.0c	38.4 ± 3.2c	33.7 ± 2.0a	0.34 ± 0.02a	3.11 ± 0.06a	1.88 ± 0.04a
60% ETc × GA_3_	81.4 ± 5.1b	72.2 ± 3.4b	11.6 ± 0.5b	0.14 ± 0.01b	1.52 ± 0.02b	0.57 ± 0.02b
60% ETc × Db-H	82.2 ± 4.7b	72.9 ± 3.3b	11.3 ± 0.6b	0.12 ± 0.00b	1.48 ± 0.02b	0.57 ± 0.01b
	**Season of 2019/2020**					
Irrigation (Ir)	*	*	**	**	**	**
100% of ETc	88.3 ± 5.1a	76.2 ± 3.7a	10.4 ± 0.4b	0.12 ± 0.00b	1.36 ± 0.09b	0.44 ± 0.02a
60% of ETc	74.5 ± 4.0b	61.0 ± 3.9b	17.8 ± 0.7a	0.20 ± 0.01a	2.18 ± 0.11a	0.88 ± 0.04a
Regulators (Re)	*	*	*	*	*	*
Control (Cn)	70.5 ± 4.3b	55.8 ± 4.0b	21.5 ± 0.9a	0.27 ± 0.02a	2.48 ± 0.15a	1.06 ± 0.06a
GA_3_	86.1 ± 4.7a	74.8 ± 4.0a	10.6 ± 0.5b	0.12 ± 0.01b	1.45 ± 0.10b	0.47 ± 0.02b
Db-H	87.7 ± 4.8a	75.3 ± 3.5a	10.4 ± 0.3b	0.11 ± 0.00b	1.37 ± 0.06b	0.46 ± 0.02b
Ir × Re	*	*	*	*	*	*
100% ETc × Cn	83.7 ± 5.1b	71.4 ± 3.3b	10.8 ± 0.3b	0.15 ± 0.01b	1.55 ± 0.08b	0.49 ± 0.03b
100% ETc× GA_3_	89.4 ± 4.8a	78.2 ± 4.1a	10.4 ± 0.5b	0.11 ± 0.00cd	1.30 ± 0.12c	0.42 ± 0.02c
100% ETc × Db-H	91.8 ± 5.4a	78.9 ± 3.8a	10.1 ± 0.3b	0.11 ± 0.00cd	1.22 ± 0.07c	0.41 ± 0.02c
60% ETc × Cn	57.2 ± 3.4c	40.1 ± 4.6c	32.1 ± 1.4a	0.38 ± 0.02a	3.41 ± 0.21a	1.62 ± 0.09a
60% ETc × GA_3_	82.8 ± 4.6b	71.3 ± 3.8b	10.7 ± 0.5b	0.12 ± 0.01c	1.60 ± 0.07b	0.52 ± 0.02b
60% ETc × Db-H	83.6 ± 4.1b	71.6 ± 3.2b	10.7 ± 0.3b	0.10 ± 0.00d	1.52 ± 0.05b	0.50 ± 0.01b

** and * indicate respectively differences at *p* ≤ 0.05 and *p* ≤ 0.01 probability level. Means followed by the same letter in each column are not significantly different according to the LSD test (*p* ≤ 0.05).

**Table 4:** Foliar application of some growth regulators (e.g., gibberellic acid; GA_3_ and diluted bee honey; Db-H) promoted osmoprotectant contents of *Vicia faba* plants grown under well watering (100% of crop evapotranspiration; ETc) or deficit irrigation (60% of ETc).

Source of Variation	Soluble Sugars (mg per g DW)	Free Proline (µM per g W)	Glycine Betaine (µM per g DW)	Total Soluble Protein (mg per g DW)
	Season of 2018/2019			
Irrigation (Ir)	*	**	**	*
100% of ETc	14.1 ± 0.3b	138.5 ± 1.8b	22.4 ± 0.4b	72.1 ± 1.5b
60% of ETc	19.9 ± 0.4a	221.5 ± 2.5a	41.4 ± 0.7a	88.6 ± 1.9a
Regulators (Re)	*	*	*	*
Control (Cn)	14.0 ± 0.3c	154.4 ± 1.6b	26.6 ± 0.5b	85.4 ± 1.8a
GA_3_	17.1 ± 0.4b	192.2 ± 2.6a	34.3 ± 0.6a	78.1 ± 1.8b
Db-H	20.1 ± 0.5a	193.5 ± 2.3a	34.9 ± 0.7a	77.6 ± 1.5b
Ir × Re	*	*	*	*
100% ETc × Cn	10.4 ± 0.2e	114.2 ± 1.5d	18.1 ± 0.3d	71.8 ± 1.5c
100% ETc× GA_3_	14.2 ± 0.4d	149.3 ± 2.0c	24.3 ± 0.4c	72.0 ± 1.7c
100% ETc × Db-H	17.8 ± 0.4c	152.1 ± 1.8c	24.8 ± 0.6c	72.4 ± 1.3c
60% ETc × Cn	17.5 ± 0.3c	194.6 ± 1.7b	35.1 ± 0.6b	98.9 ± 2.0a
60% ETc × GA_3_	19.9 ± 0.4b	235.1 ± 3.1a	44.2 ± 0.7a	84.2 ± 1.9b
60% ETc × Db-H	22.3 ± 0.5a	234.9 ± 2.8a	45.0 ± 0.7a	82.7 ± 1.7b
	**Season of 2019/2020**			
Irrigation (Ir)	*	**	**	**
100% of ETc	17.2 ± 0.4b	145.5 ± 2.2b	20.8 ± 0.3b	73.9 ± 1.6b
60% of ETc	24.8 ± 0.5a	245.5 ± 3.2a	38.5 ± 0.6a	87.6 ± 1.8a
Regulators (Re)	*	*	*	*
Control (Cn)	17.1 ± 0.4c	167.0 ± 2.5b	23.9 ± 0.4b	85.6 ± 1.8a
GA_3_	21.3 ± 0.5b	209.0 ± 3.0a	32.5 ± 0.5a	78.6 ± 1.7b
Db-H	24.6 ± 0.6a	210.6 ± 2.7a	32.6 ± 0.5a	78.1 ± 1.6b
Ir × Re	*	*	*	*
100% ETc × Cn	12.1 ± 0.3e	121.4 ± 2.0d	16.4 ± 0.2d	73.5 ± 1.7c
100% ETc× GA_3_	18.0 ± 0.5d	158.3 ± 2.5c	23.1 ± 0.4c	73.9 ± 1.6c
100% ETc × Db-H	21.4 ± 0.5c	156.8 ± 2.2c	22.8 ± 0.3c	74.2 ± 1.4c
60% ETc × Cn	22.1 ± 0.4c	212.6 ± 2.9b	31.3 ± 0.5b	97.6 ± 1.9a
60% ETc × GA_3_	24.6 ± 0.4b	259.7 ± 3.4a	41.8 ± 0.6a	83.2 ± 1.8b
60% ETc × Db-H	27.8 ± 0.6a	264.3 ± 3.2a	42.3 ± 0.6a	81.9 ± 1.8b

** and * indicate respectively differences at *p* ≤ 0.05 and *p* ≤ 0.01 probability level. Means followed by the same letter in each column are not significantly different according to the LSD test (*p* ≤ 0.05).

**Table 5:** Foliar application of some growth regulators (e.g., gibberellic acid; GA_3_ and diluted bee honey; Db-H) promoted non-enzymatic antioxidant contents of *Vicia faba* plants grown under well watering (100% of crop evapotranspiration; ETc) or deficit irrigation (60% of ETc).

Source of Variation	Ascorbate (µM per g FW)	Glutathione (µM per g FW)	α-Tocopherol (µM per g DW)	Total Phenolic Compounds (mg GAE per g DW)
	Season of 2018/2019			
Irrigation (Ir)	*	**	*	*
100% of ETc	1.59 ± 0.03b	0.88 ± 0.02b	2.22 ± 0.04b	8.10 ± 0.27b
60% of ETc	2.28 ± 0.04a	1.49 ± 0.03a	3.10 ± 0.05a	10.08 ± 0.32a
Regulators (Re)	*	*	*	*
Control (Cn)	1.69 ± 0.03c	0.98 ± 0.02c	2.39 ± 0.04c	10.27 ± 0.35a
GA_3_	1.99 ± 0.04b	1.22 ± 0.03b	2.72 ± 0.04b	8.86 ± 0.28b
Db-H	2.14 ± 0.04a	1.36 ± 0.03a	2.88 ± 0.05a	8.15 ± 0.26c
Ir × Re	*	*	*	*
100% ETc × Cn	1.23 ± 0.02e	0.64 ± 0.01e	1.89 ± 0.03e	8.12 ± 0.30c
100% ETc× GA_3_	1.64 ± 0.03d	0.89 ± 0.02d	2.24 ± 0.04d	8.10 ± 0.26c
100% ETc × Db-H	1.91 ± 0.03c	1.11 ± 0.02c	2.53 ± 0.05c	8.09 ± 0.24c
60% ETc × Cn	2.14 ± 0.04b	1.32 ± 0.03b	2.88 ± 0.05b	12.42 ± 0.39a
60% ETc × GA_3_	2.33 ± 0.04a	1.55 ± 0.04a	3.19 ± 0.04a	9.62 ± 0.30b
60% ETc × Db-H	2.36 ± 0.04a	1.60 ± 0.03a	3.23 ± 0.05a	8.21 ± 0.27c
	**Season of 2019/2020**			
Irrigation (Ir)	**	**	*	*
100% of ETc	1.47 ± 0.02b	0.80 ± 0.01b	2.39 ± 0.05b	7.88 ± 0.20b
60% of ETc	2.26 ± 0.05a	1.43 ± 0.03a	3.37 ± 0.07a	9.85 ± 0.25a
Regulators (Re)	*	*	*	*
Control (Cn)	1.58 ± 0.03c	0.95 ± 0.02c	2.56 ± 0.05c	10.05 ± 0.27a
GA_3_	1.93 ± 0.04b	1.16 ± 0.02b	2.94 ± 0.06b	8.68 ± 0.23b
Db-H	2.09 ± 0.05a	1.25 ± 0.02a	3.15 ± 0.07a	7.88 ± 0.18c
Ir × Re	*	*	*	*
100% ETc × Cn	1.19 ± 0.01e	0.66 ± 0.01e	1.98 ± 0.04e	7.89 ± 0.21c
100% ETc× GA_3_	1.44 ± 0.02d	0.79 ± 0.01d	2.41 ± 0.04d	7.91 ± 0.19c
100% ETc × Db-H	1.79 ± 0.04c	0.94 ± 0.01c	2.79 ± 0.06c	7.85 ± 0.20c
60% ETc × Cn	1.97 ± 0.04b	1.23 ± 0.02b	3.14 ± 0.06b	12.20 ± 0.32a
60% ETc × GA_3_	2.41 ± 0.05a	1.52 ± 0.03a	3.47 ± 0.07a	9.44 ± 0.26b
60% ETc × Db-H	2.39 ± 0.06a	1.55 ± 0.03a	3.51 ± 0.08a	7.91 ± 0.16c

** and * indicate respectively differences at *p* ≤ 0.05 and *p* ≤ 0.01 probability level. Means followed by the same letter in each column are not significantly different according to the LSD test (*p* ≤ 0.05).

**Table 6:** Foliar application of some growth regulators (e.g., gibberellic acid; GA_3_ and diluted bee honey; Db-H) promoted antioxidant enzyme activities of *Vicia faba* plants grown under well watering (100% of crop evapotranspiration; ETc) or deficit irrigation (60% of ETc).

Source of Variation	Superoxide Dismutase (A_564_ per min per g Protein)	Catalase (A_290_ per min per g Protein)	Glutathione Reductase (A_340_ per min per g Protein)	Ascorbate Peroxidase (A_290_ per min per g Protein)
	Season of 2018/2019			
Irrigation (Ir)	**	**	**	*
100% of ETc	15.5 ± 0.2b	56.5 ± 0.7b	23.4 ± 0.3b	68.4 ± 0.8b
60% of ETc	23.4 ± 0.4a	85.5 ± 0.8a	37.1 ± 0.4a	93.2 ± 0.7a
Regulators (Re)	*	*	*	*
Control (Cn)	16.7 ± 0.3b	63.4 ± 0.7b	26.6 ± 0.3b	71.6 ± 0.7b
GA_3_	20.9 ± 0.3a	75.3 ± 0.8a	32.2 ± 0.4a	85.4 ± 0.8a
Db-H	20.8 ± 0.3a	74.3 ± 0.8a	32.1 ± 0.4a	85.4 ± 0.7a
Ir × Re	*	*	*	*
100% ETc × Cn	14.1 ± 0.2d	48.5 ± 0.6d	19.8 ± 0.2d	62.1 ± 0.8d
100% ETc× GA_3_	16.4 ± 0.2c	61.2 ± 0.8c	25.1 ± 0.3c	71.6 ± 0.8c
100% ETc × Db-H	16.0 ± 0.2c	59.8 ± 0.7c	25.4 ± 0.3c	71.4 ± 0.7c
60% ETc × Cn	19.2 ± 0.4b	78.3 ± 0.8b	33.3 ± 0.3b	81.1 ± 0.6b
60% ETc × GA_3_	25.4 ± 0.3a	89.4 ± 0.8a	39.2 ± 0.5a	99.2 ± 0.8a
60% ETc × Db-H	25.6 ± 0.4a	88.7 ± 0.9a	38.8 ± 0.5a	99.3 ± 0.7a
	**Season of 2019/2020**			
Irrigation (Ir)	**	**	**	*
100% of ETc	17.3 ± 0.3b	52.9 ± 0.5b	24.2 ± 0.3b	64.5 ± 0.7b
60% of ETc	27.4 ± 0.4a	80.1 ± 0.8a	38.4 ± 0.5a	85.4 ± 1.0a

Regulators (Re)	*	*	*	*
Control (Cn)	20.1 ± 0.3b	58.3 ± 0.7b	27.1 ± 0.4b	69.2 ± 0.8b
GA_3_	23.4 ± 0.4a	70.7 ± 0.8a	33.8 ± 0.5a	77.9 ± 0.9a
Db-H	23.7 ± 0.4a	70.7 ± 0.6a	33.1 ± 0.4a	78.0 ± 0.8a
Ir × Re	*	*	*	*
100% ETc × Cn	15.6 ± 0.2d	44.2 ± 0.5d	21.0 ± 0.3d	59.7 ± 0.7d
100% ETc× GA_3_	18.1 ± 0.3c	57.1 ± 0.6c	26.2 ± 0.3c	66.8 ± 0.6c
100% ETc × Db-H	18.3 ± 0.3c	57.4 ± 0.5c	25.4 ± 0.2c	67.1 ± 0.7c
60% ETc × Cn	24.5 ± 0.3b	72.3 ± 0.8b	33.1 ± 0.4b	78.6 ± 0.9b
60% ETc × GA_3_	28.7 ± 0.4a	84.2 ± 0.9a	41.3 ± 0.6a	88.9 ± 1.1a
60% ETc × Db-H	29.0 ± 0.5a	83.9 ± 0.7a	40.7 ± 0.5a	88.8 ± 0.9a

** and * indicate respectively differences at *p* ≤ 0.05 and *p* ≤ 0.01 probability level. Means followed by the same letter in each column are not significantly different according to the LSD test (*p* ≤ 0.05).

**Table 7:** Foliar application of some growth regulators (e.g., gibberellic acid; GA_3_ and diluted bee honey; Db-H) promoted nutrient contents of *Vicia faba* plants grown under well watering (100% of crop evapotranspiration; ETc) or deficit irrigation (60% of ETc).

Source of Variation	Nitrogen (mg per g Dry Weight)	Phosphorus (mg per g Dry Weight)	Potassium (mg per g Dry Weight)	Iron (mg per g Dry Weight)	Manganese (mg per g Dry Weight)	Zinc (mg per g Dry Weight)
	Season of 2018/2019					
Irrigation (Ir)	*	*	*	*	*	*
100% of ETc	19.3 ± 1.2a	2.51 ± 0.14a	19.0 ± 1.3a	0.77 ± 0.03a	0.50 ± 0.01a	0.33 ± 0.01a
60% of ETc	15.3 ± 1.3b	1.95 ± 0.10b	16.3 ± 1.0b	0.61 ± 0.01b	0.41 ± 0.01b	0.26 ± 0.01b
Regulators (Re)	*	*	*	*	*	*
Control (Cn)	14.5 ± 1.1c	1.81 ± 0.10c	14.6 ± 1.0c	0.59 ± 0.02c	0.38 ± 0.01c	0.24 ± 0.00c
GA_3_	18.2 ± 1.2b	2.37 ± 0.11b	18.2 ± 1.1b	0.71 ± 0.02b	0.48 ± 0.01b	0.31 ± 0.01b
Db-H	19.4 ± 1.5a	2.51 ± 0.15a	20.3 ± 1.5a	0.77 ± 0.03a	0.52 ± 0.02a	0.35 ± 0.01a
Ir × Re	*	*	*	*	*	*
100% ETc × Cn	16.8 ± 0.9c	2.10 ± 0.12c	17.2 ± 1.1c	0.68 ± 0.02c	0.44 ± 0.01c	0.29 ± 0.00c
100% ETc× GA_3_	19.7 ± 1.2b	2.56 ± 0.12b	18.9 ± 1.2b	0.76 ± 0.02b	0.49 ± 0.01b	0.33 ± 0.01b
100% ETc × Db-H	21.5 ± 1.5a	2.88 ± 0.17a	20.9 ± 1.7a	0.88 ± 0.04a	0.57 ± 0.02a	0.38 ± 0.01a
60% ETc × Cn	12.1 ± 1.3d	1.52 ± 0.07d	11.9 ± 0.8d	0.50 ± 0.01d	0.31 ± 0.00d	0.18 ± 0.00d
60% ETc × GA_3_	16.7 ± 1.2c	2.18 ± 0.10c	17.4 ± 0.9c	0.66 ± 0.01c	0.46 ± 0.01c	0.28 ± 0.01c
60% ETc × Db-H	17.2 ± 1.4c	2.14 ± 0.12c	19.6 ± 1.2b	0.66 ± 0.01c	0.47 ± 0.01c	0.31 ± 0.01b
	**Season of 2019/2020**					
Irrigation (Ir)	*	*	*	*	*	*
100% of ETc	20.2 ± 0.9a	2.41 ± 0.11a	20.9 ± 1.0a	0.80 ± 0.02a	0.57 ± 0.01a	0.36 ± 0.00a
60% of ETc	15.9 ± 0.6b	1.84 ± 0.09b	15.9 ± 0.8b	0.65 ± 0.01b	0.44 ± 0.00b	0.29 ± 0.00b
Regulators (Re)	*	*	*	*	*	*
Control (Cn)	14.7 ± 0.6c	1.70 ± 0.07c	14.8 ± 0.8c	0.61 ± 0.01c	0.41 ± 0.00c	0.27 ± 0.00c
GA_3_	18.9 ± 0.7b	2.22 ± 0.11b	18.9 ± 1.0b	0.75 ± 0.02b	0.53 ± 0.01b	0.34 ± 0.00b
Db-H	20.7 ± 1.0a	2.45 ± 0.13a	21.6 ± 1.0a	0.83 ± 0.02a	0.58 ± 0.01a	0.37 ± 0.01a
Ir × Re	*	*	*	*	*	*
100% ETc × Cn	17.4 ± 0.8c	1.98 ± 0.09c	16.9 ± 0.9c	0.71 ± 0.01c	0.50 ± 0.00c	0.31 ± 0.00c
100% ETc× GA_3_	19.9 ± 0.8b	2.42 ± 0.11b	20.4 ± 0.9b	0.78 ± 0.02b	0.56 ± 0.01b	0.36 ± 0.00b
100% ETc × Db-H	23.4 ± 1.1a	2.83 ± 0.14a	25.3 ± 1.2a	0.91 ± 0.02a	0.64 ± 0.01a	0.42 ± 0.01a
60% ETc × Cn	11.9 ± 0.4d	1.42 ± 0.05d	12.6 ± 0.6d	0.50 ± 0.00d	0.32 ± 0.00d	0.22 ± 0.00d
60% ETc × GA_3_	17.8 ± 0.6c	2.02 ± 0.10c	17.4 ± 1.0c	0.72 ± 0.01c	0.49 ± 0.00c	0.32 ± 0.00c
60% ETc × Db-H	17.9 ± 0.9c	2.07 ± 0.12c	17.8 ± 0.8c	0.74 ± 0.01bc	0.51 ± 0.01c	0.32 ± 0.00c

** and * indicate respectively differences at *p* ≤ 0.05 and *p* ≤ 0.01 probability level. Means followed by the same letter in each column are not significantly different according to the LSD test (*p* ≤ 0.05).

**Table 8:** Foliar application of some growth regulators (e.g., gibberellic acid; GA_3_ and diluted bee honey; Db-H) promoted plant hormonal contents of *Vicia faba* plants grown under well watering (100% of crop evapotranspiration; ETc) or deficit irrigation (60% of ETc).

Source of Variation	Indole-3-Acetic Acid (µg per g FW)	Gibberellic Acid (µg per g FW)	Cytokinins (µg per g FW)	Abscisic Acid (µg per g FW)
	Season of 2018/2019			
Irrigation (Ir)	*	*	*	*
100% of ETc	18.1 ± 0.15a	33.1 ± 0.29a	24.6 ± 0.18a	4.23 ± 0.05b
60% of ETc	14.2 ± 0.14b	25.1 ± 0.26b	18.4 ± 0.16b	6.29 ± 0.06a
Regulators (Re)	*	**	*	*
Control (Cn)	12.2 ± 0.10c	18.5 ± 0.20c	15.5 ± 0.13c	7.43 ± 0.07a
GA_3_	16.0 ± 0.15b	41.9 ± 0.40a	22.0 ± 0.18b	4.45 ± 0.05b
Db-H	20.4 ± 0.19a	26.9 ± 0.23b	27.2 ± 0.21a	3.91 ± 0.05c
Ir × Re	*	*	*	*
100% ETc × Cn	14.1 ± 0.11c	22.4 ± 0.19d	18.7 ± 0.15c	5.22 ± 0.06b
100% ETc× GA_3_	17.4 ± 0.15b	45.6 ± 0.39a	25.4 ± 0.20b	3.77 ± 0.04e
100% ETc × Db-H	22.9 ± 0.19a	31.2 ± 0.28c	29.8 ± 0.20a	3.69 ± 0.04e
60% ETc × Cn	10.3 ± 0.09d	14.6 ± 0.20e	12.2 ± 0.11d	9.64 ± 0.07a
60% ETc × GA_3_	14.5 ± 0.15c	38.2 ± 0.41b	18.5 ± 0.15c	5.12 ± 0.06c
60% ETc × Db-H	17.9 ± 0.18b	22.6 ± 0.18d	24.6 ± 0.22b	4.12 ± 0.05d
	**Season of 2019/2020**			
Irrigation (Ir)	*	*	*	**
100% of ETc	20.4 ± 0.18a	33.9 ± 0.29a	24.2 ± 0.20a	3.75 ± 0.04b
60% of ETc	15.8 ± 0.14b	24.7 ± 0.22b	18.2 ± 0.19b	6.29 ± 0.07a
Regulators (Re)	*	**	*	*
Control (Cn)	14.1 ± 0.13c	17.6 ± 0.20c	15.0 ± 0.14c	7.47 ± 0.08a
GA_3_	18.1 ± 0.15b	44.4 ± 0.37a	20.9 ± 0.19b	4.19 ± 0.05b
Db-H	22.3 ± 0.20a	26.0 ± 0.21b	27.7 ± 0.26a	3.40 ± 0.04c
Ir × Re	*	*	*	*
100% ETc × Cn	16.8 ± 0.18c	21.6 ± 0.22d	17.6 ± 0.12c	4.98 ± 0.05b
100% ETc× GA_3_	19.7 ± 0.17b	50.2 ± 0.45a	23.8 ± 0.25b	3.48 ± 0.03c
100% ETc × Db-H	24.8 ± 0.19a	29.8 ± 0.21c	31.2 ± 0.22a	2.78 ± 0.03d
60% ETc × Cn	11.3 ± 0.08d	13.6 ± 0.18e	12.4 ± 0.15d	9.96 ± 0.11a
60% ETc × GA_3_	16.4 ± 0.12c	38.5 ± 0.29b	18.0 ± 0.13c	4.89 ± 0.06b
60% ETc × Db-H	19.7 ± 0.21b	22.1 ± 0.20d	24.2 ± 0.30b	4.01 ± 0.04c

** and * indicate respectively differences at *p* ≤ 0.05 and *p* ≤ 0.01 probability level. Means followed by the same letter in each column are not significantly different according to the LSD test (*p* ≤ 0.05).

**Table 9:** Some initial physical and chemical soil properties.

Layer (cm)	Particle Size Distribution				Bulk Density (g cm^−3^)	K_sat_ Cm h^−1^	FC (%)	WP (%)	AW (%)	pH	ECe (dS.m^−1^)	OM (%)	CaCO_3_ (%)
	Sand %	Silt %	Clay %	TC									
0–30	20	38	42	CL	1.40	1.2	34.3	19.7	14.6	7.76	2.85	1.50	4.3
30–60	17	37	46	CL	1.36	0.9	32.2	19.1	13.1	7.75	2.98	1.10	4.2

TC = Texture class, CL = Clay loam, FC = Field capacity, WP = Wilting point, AW = Available water, OM = Organic matter, and K_sat_ = Hydraulic conductivity.

**Table 10:** Physico-chemical composition of raw clover honey (on a fresh weight basis).

Property/Component	Unit	Value
Moisture	%	16.8
Proteins		0.28
Organic acids		0.48
pH		4.14
**Osmoprotectants:**		
Proline	mg kg^−1^ FW	47.8
Total soluble sugars	%	82.6
Amino acids		0.33
**Sugar fractions:**		
Fructose	%	44.2
Glucose		25.9
Maltose		3.7
Sucrose		4.21
**Mineral nutrients:**		
Potassium (K)	mg kg^−1^ FW	456.8
Phosphorus (P)		50.2
Magnesium (Mg)		84.2
Calcium (Ca)		71.4
Sulphur (S)		77.8
Iron (Fe)		69.8
Manganese (Mn)		8.4
Zinc (Zn)		5.5
Copper (Cu)		4.6
Iodine (I)		81.4
Sodium (Na)		42.9
Selenium (Se)		0.92
**Antioxidants and Vitamins:**		
Ascorbic acid (vitamin C)	mg kg^−1^ FW	24.2
Thiamine (B1)		0.14
Riboflavin (B2)		0.18
Niacin (B3)		1.67
Pantothenic acid (B5)		1.08
Pyridoxine (B6)		2.27
Folate (B9)		0.21
DPPH radical-scavenging activity	%	88.2

## Data Availability

The data presented in this study are available upon request from the corresponding author.

## Supplementary Materials

The following are available online at https://www.mdpi.com/article/10.3390/plants10040748/s1, Table S1: A preliminary pot study conducted to identify the optimal concentration of diluted bee-honey (Db-H) and gibberellic acid (GA_3_), as well as identifying the drought threshold of faba bean (Giza 40 cultivar) for the main study.

## Sample Availability

Samples of faba bean plant are available from the authors.
