# Supplementary material for: Exogenous Gibberellic Acid or Dilute Bee Honey Boosts Drought Stress Tolerance in Vicia faba by Rebalancing Osmoprotectants, Antioxidants, Nutrients, and Phytohormones
Source: Plants (Basel). 2021 Apr 11;10(4):748. doi: 10.3390/plants10040748 (PMC8068922; doi:10.3390/plants10040748)
Supplement: Supplementary file 1 [file plants-10-00748-s001.pdf]

Table S1. A preliminary pot study conducted to identify the optimal concentration of diluted bee-honey (Db-H) and gibberellic acid (GA_3), as well as identifying the drought threshold of faba bean (Giza 40 cultivar) for the main study.

Treat-ments	No. of Leaves Plant⁻¹	Leaf Area Plant⁻¹ (cm²)	Shoot DW Plant⁻¹ (g)	No. of Green Pods Plant⁻¹	Total Chlorophylls (mg g⁻¹ FW)
Control	18.8±2.0c	76.1±8.3c	8.2±0.7c	9.7±1.1c	2.22±0.05c
10 g Db-H/L	21.1±2.2b	89.5±8.9b	10.1±1.2b	12.1±1.5b	2.48±0.07b
**20 g Db-H/L**	**25.4±2.8a**	**107.5±11.1a**	**13.4±1.9a**	**16.2±1.8a**	**2.79±0.11a**
30 g Db-H/L	24.9±2.5a	108.4±10.9a	13.4±1.8a	16.3±1.7a	2.82±0.11a
Control	18.8±2.0c	76.1±8.3c	8.2±0.7c	9.7±1.1c	2.22±0.05c
10 mg GA_3/L	20.8±2.1b	84.7±9.5b	10.0±0.9b	11.3±1.3b	2.42±0.05b
**20 mg GA_3/L**	**23.4±2.4a**	**101.6±10.9a**	**12.4±1.4a**	**14.6±1.5a**	**2.69±0.09a**
30 mg GA_3/L	23.6±2.5a	104.5±11.1a	12.6±1.6a	14.8±1.6a	2.68±0.09a
100% of ETc	18.8±2.0a	76.1±8.3a	8.2±0.7a	9.7±1.1a	2.22±0.05a
80% of ETc	14.4±1.5b	52.4±6.0b	6.9±0.5b	7.1±0.8b	1.56±0.04b
**60% of ETc**	**4.9±0.4c**	**12.8±1.3c**	**2.6±0.2c**	**2.3±0.3c**	**0.69±0.02c**
40% of ETc	Plants died				

The same letters with mean values±SE in each column indicate non-significant differences, and different letters indicate significant differences according to LSD test ($P \leq 0.05$).

It is evident from this Table (S1) that the best concentration of diluted bee-honey (Db-H at 20g per liter) and gibberellic acid (GA_3 at 20 mg per liter) was chosen, which gave the best results from an economic point of view. As for the deficit irrigation water treatments, the deficit irrigation treatment at 60% of ETc was chosen prior to the treatment in which the plants died.
